# Abstracts and Gleanings

**Published:** 1885-07-20

**Authors:** 


### ABSTRACTS AND GLEANINGS.

**Hemorrhagic Malarial Fever.**—Dr. Jerome Cochrane, of Alabama, read at American Medical Association a short paper on Hemorrhagic Malarial Fever, and stated some of his conclusions as follows :

1. This form of malarial fever oiiginates only in malarial regions, though it may exhibit itself elsewhere.

2. The poison is the same as that in remittent and intermittent fevers.

3. It may show itself in the following form : *a*, intermittent ; *b*, remittent ; *c*, congestive ; *d*, quasi-continued.

4. The congestive form is almost necessarily fatal ; the bad ; the intermittent form is less fatal.

5: The negro is comparatively exempt.

6. Only those persons suffering under malarial cachexia are attacked.

7. One attack is not protective.

8. It begins usually in the afternoon or at night, with a chill, followed by fever, bilious vomiting, discolored urine, jaundice.

9. The fever, except in the remittent and quasi-continued form, is not usually high.

10. Skin harsh and dry.

11. Bowels constipated, liver torpid.

12. Vomiting, early nausea, matter yellow, green, black, or even blue.

13. The black vomit is usually changed to bile, and in the beginning of the case.

14. Characteristic red urine, profuse at first, begins with chill. In fatal cases, there is suppression.

15. This red urine is albuminous with granular casts. Color is due to blood-pigment hæmoglobin, not corpuscles.

16. The post-mortem appearances are similar to those of other malarial affections, with enlargement of the kidneys added.

| | |
|---|---|
| Cases reported | 642 |
| Deaths | 188 |
| Per cent | 24.60 |

Blood-corpuscles in the urine are accidental. The serum of blisters is colored the same way.

As to treatment :

1. The superabundance of bile renders the use of mercurials of great service. Some physicians use small, frequently repeated doses. Others like large doses ; in one instance, a doctor was in the habit of givıng 60 to 70 grains at a time.

2. Warm drinks are of service in promoting emesis, to get rid of the large amount of bile thrown into the stomach.

3. Sweating to reduce the harshness of the skin, and promote its action ; for this purpose, heat to the surface, hot drinks, and occasionally diaphoretics are used.

4. Management of the kidneys is a disputed point. The majority are in favor of letting them alone. Even in the suppression of urine, trust to the compensatory action of the bowels and skin.

5. Quinine was formerly looked upon as the sheet-anchor in this affection. It exerts less control here than in any other form of malaria, and is losing ground.

Dr. Ghent, of Texas, had treated forty-seven cases, with five deaths. He thinks the red color due to blood corpuscles. He uses calomel first, last, and always.

Dr. Taylor, U. S. Army, had an experience similar to Dr. Cochrane's. But he had always found micrococci in the blood of these cases, and always blood-corpuscles and albumen in the urine. It is very fatal. Quinine will control the fever to a certain extent, but nothing more. Calomel and bicarbonate of soda in small doses are of great value.

Dr. Joseph Jones, of New Orleans, thinks some cases of so-called hemorrhagic malarial fever, or hæmaturia, are really melanuria. The fibrin is increased as in inflammations. There is red matter in the urine in all fevers. In melanuria, in addition to blood-corpuscles, there is also bile, as proved by testing with nitric acid. Serum of a blister is so colored. The origin of this disease is congestion of the kidneys, and the consequent pouring out of blood in the tubuli, just as in congestion of the brain. The rest of the pathology is due to the want of functioning of the kidneys because of this fact. The prime cause is malaria. He is prepared for good effect from calomel, since the pathology includes an increase of fibrin in the blood ; but he would not abandon quinine.

**The Therapeutical Value of Arsenic in Anæmia and Atrophic Conditions.**—Dr. Samuel Wilkes, in the *Lancet* for April 11, writes strongly in favor of arsenic in many diseases where skepticism as to its use on the part of a large portion of the profession has generally prevailed. There can be no doubt that many of the cutaneous affections cured by arsenic have a gouty origin, and therefore it is not surprising that the same remedy has a great power in preventing attacks of gout. Then, this gouty class of persons are often neuralgic, and it may be in them especially that arsenic is the best nervine remedy. He has found it amongst the most efficacious medicines, and in some cases the only remedy. Thus, before the introduction of nitrite of amyl and glonoine for agina pectoris, he relied mainly on arsenic, and in some cases kept off attacks for weeks where they had previously occurred almost daily. But the most remarkable effects of this remedy are seen in anæmia and various forms of cachexia and atrophy. One case which he cites was a lady about forty years of age, who was pronounced to be the subject of idiopathic anæmia. Her bloodless and feeble condition compelled her to keep her bed, and it was never believed that she would rise from it again. Arsenic was used, she soon began to improve, and in a few weeks was able to visit her doctor at his house. Her husband was not surprised at the action of the remedy, for, as he said, if he had a horse which was not "thrifty" he gave it arsenic, rendering it again plump and glossy. Another case of the so-called pernicious anæmia was in a gentleman who had gradually grown anæmic and breathless, so as to be unable to leave his house, and he walked with much difficulty. He took five drops of liquor arsenicalis, and in a month he was comparatively well. In most of the cases where arsenic has succeeded, iron had previously failed. It is, however, in wasting and general cachexia that Dr. Wilkes has been most pleased with its action. He details several cases where there were evidences of extreme wasting and debility, attributable to no special disease, and where arsenic effected cures.

He has never given very large doses, generally four or five drops of the liquor arsenicalis three times a day, or a little more of the soda preparation ; nor has he observed any injurious effects from its long use, although, as is known, it becomes absorbed into the system, the urine showing its presence many weeks after its administration has ceased.

An editorial on this article, in the same number of the *Lancet*, considers Dr. Wilkes' testimony of great value, as coming from one who is far too much imbued with scientific caution to lavish undeserved credit on any pharmacopœial preparation. The testimony of Dr. Wilkes on its efficacy in idiopathic anæmia is borne out by the experience of many physicians ; among the most recent being Dr. Warfinge, of Stockholm, who reported several cases of remarkably rapid arrest of the downward progress of the disease, and even of recovery, under the use of arsenic. All such cases should, however, be subjected to prolonged supervision, as it is notorious that relapses are prone to occur. The same remedy has been also successfully employed in an even more definite cachexia, viz.: Hodgkins' disease, where the administration of arsenic has been supplemented by its injection into the hyperplastic lymphatic glands with, according to Winiwater, astonishing results.—*Jour. Am. Ass.*

**Summer Complaints of Children**.—A writer in Southern Clinic says : "Well, now, my young friends who read this, I will ask you to allow a little egotism ; but I think I can give you the best treatment for cholera infantum ever written, and in the fewest words :

1st. Hot or warm bath, grain doses of calomel and saccharated pepsin or lactopeptine on crushed ice every fifteen or twenty minutes.

2d. After the stomach and bowels have been thoroughly emptied, if the little patient is threatened with collapse, stimulate and control the bowel and stomach with opium. When the first onslaught is over, a great many children will have diarrhœa for days and weeks, and the too common error has been among book-wise practitioners, that this condition is chronic cholera infantum, and they continue to treat this trouble with calomel ! calomel ! ! calomel ! ! ! vainly waiting to see the stools change color, and hoping that when the portal congestion is relieved the diarrhœa will cease. Sometimes this plan succeeds, but in far too many cases a large per centage of cases die from continued dosing with calomel and chalk, when a prompt astringent would end the whole trouble, give the bowels rest, and under appropriate diet, the child would go on to a fair recovery. There is no more reason why a child should be allowed to have diarrhœa for weeks, than an adult. There is no danger in checking up the bowels, and it should be done.

I have noticed that there seems to be a morbid fear among many practitioners about controlling the diarrhœa of infants, particularly that form which follows almost constantly after an attack of cholera infantum. This is an error.

I must put in another plea for the life of these little patients while I have a chance. It is this:

Many children when attacked by cholera infantum are allowed to die for want of water. We have all seen the collapse of cholera in the adult and know that the great cry of these patients is for water: the sunken eye, the flabby skin and husky voice, all tell us that these poor patients are in need of water; the blood has become thick and can scarcely circulate and unless the fluid is supplied the patient will surely die, if not from cholera, for the want of water.

I have so often seen exactly this state of affairs in infants, that I regard them as analogous cases, and often when all other remedies have failed me, I have been able to check vomiting and purging and restore the skin to its proper, soft, moist and velvety condition by the free use of iced water, given to these little sufferers *ad libitum.*

**The Treatment of Carbuncle Without Incision.**—In the course of the paper on this subject before the American Medical Association, by Dr. L. Duncan Bulkley (Med. News, May 9, '85), the author related the case of a gentleman, aged 56, large and florid, who suffered for several years with eczema of the left foot. He was also diabetic. Following upon this eruption was a large carbuncle. He applied to this tumor, thickly spread on the woolen side of lint, the following ointment:

℞. Ergot. fl. ext. . . . . . . . . . . . . . . . . . . . . . . . . . . . . . . ʒ ij

Zinc oxidi . . . . . . . . . . . . . . . . . . . . . . . . . . . . . . ʒ ss

Unguent. aq. rosæ. . . . . . . . . . . . . . . . . . . . . . . . . . ʒ ij.

M.

Covering this was cotton-batting, to prevent blows or injury. He was given sulphite of calcium ¼ gr. every two hours, and occasionally the following:

℞. Magnesia sulphat. . . . . . . . . . . . . . . . . . . . . . . . . . . . ʒ iv

Ferri sulphat. . . . . . . . . . . . . . . . . . . . . . . . . . . . . . ʒ j

Acid. sulphuric dil. . . . . . . . . . . . . . . . . . . . . . . . . . . ʒ iij

Syr. zingiber. . . . . . . . . . . . . . . . . . . . . . . . . . . . . . ℥ j

Aquæ. . . . . . . . . . . . . . . . . . . . . . . . . . . . . . . . . . . ℥ iij.

M. S. Teaspoonful in water through a tube three times daily.

At bedtime Dover's powder was administered, to give rest when required. The result of the treatment was cessation of pain, rapid resolution, and a cure, except some induration, in eighteen days. The man continued at work.

He summed up his paper as follows :

1. Avoid any irritation, as pressure or blows, etc.
2. Avoid warmth and moisture, as in poultices.
3. Avoid incisions.
4. Do not use stimulants.
5. Protect the inflamed parts with the ointment given above. The solid extract of ergot may be used if desired. Spread the ointment at least one-third inch thick.
6. Use sulphite of calcium every two hours for its effect upon suppuration.
7. Employ good, nutritious food, and fresh air.
8. A sedative, if desired, and occasionally the laxative and refrigerant tonic as above.

The advantages are :

1. Short time required for recovery.
2. Cessation of pain.
3. No scar.
4. No operation.
5. No detention from business.—*Med. and Surg. Reporter.*

**Fighting Cholera by Inoculation.**—We learn from a Madrid telegram that the Spanish Government has granted permission to inoculate people with cholera virus.

Dr. Jaime Ferran, the discoverer of the process, is stated to have operated, up to the present time, on 8,000 persons in the province of Valencia alone. Two well known Madrid doctors, Senors Moreno and Tolosa, who went to study Dr. Ferran's discovery, were inoculated by him. Four hours afterward they felt all the symptoms of cholera—coldness, cramps, diarrhœa, fever, and delirium—but after sixteen hours they were all right again. At the Aleira Hospital all the inmates were inoculated by Dr. Ferran excepting two, who refused to submit to the operation. Cholera attacked these two and they died of it, while all the others were safe. The same thing occurred elsewhere. Delegations from all parts of the country are going to study the alleged discovery, and the Cortes has voted a sum to enable Dr. Ferran to prosecute his experiments, as he is poor.

This possible successor of Jenner is said to be only thirty-three years old. Born in Tarragona, he studied medicine in Tortosa, and took his degree at Barcelona.

He claims to have been led to his discovery by following the cholera microbe through its various stages of development and transformation until he detected a spore (the *peronospora Ferran*), which, in his belief, contains the real virus of cholera. It was with specimens of this organism that he made his inoculative substance.

Thus far, what has been demonstrated appears to be simply the fact that the process results in a modified form of cholera. Judging, however, from the latest news relative to the progress of the malady in Spain, it seems certain that the actual value of Dr. Ferran's theory will soon be put to the test. If, when the plague has spent its force, it shall be ascertained that, of the many thousands whom he has inoculated in a single province, not one, or but a very small percentage, has contracred the disease—although all of them were equally exposed with the rest of the community—the utility of his procedure might be regarded as established—so far as single visitations were concerned. But since cholera, according to the general belief, may be taken an indefinite number of times, it would seem that inoculation cannot set up any protective changes in the system, as vaccination does in the case of small-pox. The process could only act as a preventive once, and would have to be repeated with every fresh appearance of the epidemic. This objection, while, of course, not altogether a fatal one, may suffice to take inoculation out of the category of practical resources against the disease.

To determine finally whether the artificial protection of cholera affords a permanent or only a temporary protection, would evidently require a long time, and an extended series of carefully-conducted experiments. Meanwhile, whatever enthusiastic reports may be wafted to our ears, it will be unsafe to rely implicity upon the new process.—*Medical Times.*

**Hints on the Usage of Drainage Tubes.**—Dr. W. L. Getz thus writes in the Journal Am. Med. Ass., July 3, 1885: Some months ago we had occasion to evacuate a pelvic abcess, and use a drainage tube as a means of thorough drainage. Not having at hand at the time a regular drainage tube, we constructed one out of a piece of plain (small size) rubber tubing. After being in the opening for several days we desired to replace it by another tube; we attempted to remove it, found that the opening in the tissues through which the tube had passed had contracted so as to hold tightly the tube; and although we made but slight traction, anticipating the possibility of the tube's breaking, to our extreme discomfort and dissatisfaction, we soon realized that our anticipations were realities, a portion of the tube, an inch in length, remaining within the pelvic cavity.

We succeeded in removing it by dilating the opening through which the tube was passed; then introducing a small blunt hook, we succeeded in drawing the piece of tube into position, so that it was easily grasped by a pair of forceps and extracted, much to our satisfaction, and with a vow that in the future we shall select with caution our material for drainage tubes.

A hint on the removal of tubes and upon their introduction may not be out of order here, under circumstances as above described; where the tissues firmly hold the tube, we should adopt the plan of inserting within the tube a dilator of some kind with which to dilate the tissues before we attempt to withdraw the tube.

As a satisfactory method of introducing drainage tubes, we have found that where a trocar and canula were necessary to evacuate the contents of a cyst or an abscess, that by taking the precaution to use a canula a trifle larger than the drainage tube to be used, the latter could be conveniently passed through the canula into position and then the canula withdrawn.—*Quarterly Comp.*

**Goitre Successfully Treated.**—The Medical Record, Jan. 10, 1885, says Dr. G. W. McCaskey, of Fort. Wayne, Ind., was consulted on July 2, 1884, by a young woman, unmarried, aged twenty-five, on account of a small goitre. It measured three and one-half inches from side to side and from one and one-half to two and one-half inches vertically, being larger on the right side. It was firm and non-cystic.

The treatment consisted of Lugol's solution, gtt vj., three times per day. From once to twice each week the tumor was injected with tr. iodine, gtt. iv.–vj., the length of time between injections depending upon the persistence of the swelling. About twice each week the galvanic current (twelve cells), with the cathode over the tumor, was used for twenty to thirty minutes.

The result of three months' treatment was to cause the entire disappearance of the enlargement upon the left side, leaving what seemed to be a slight thickening and induration of the tissues on the right side. This thickening had remained up to date of last examination, some two months after the cessation of active treatment. The neck was reduced in size from thirteen and one-half to eleven inches.

The patient lost fifteen pounds in weight, about one-seventh her entire weight. When the debility became very marked, Lugol's solution was suspended for a week. This was necessary twice during the treatment. When the treatment was discontinued the patient regained her original weight in less than a month, without any recurrence of the tumor.

The troublesome dysphonia, which led the patient to consult me, disappeared in about one month after the commencement of treatment. The pressure seemed to be removed from the larynx before any obvious diminution had occurred in the bulk of the tumor.—*Quarterly Comp.*

**Medical Prizes.**—The Societe Protectrice de l'Enfance de Lyon offers two prizes, to be awarded in 1886 and 1887, respectively, for the best essays on the following subjects: 1. On the different methods of vaccination; the age at which it is proper to vaccinate; and the prejudices against vaccination which are to be overcome. 2. On the influence of maternal occupation upon the fecundity of women, the course of pregnancy, and the vitality and health of the infants. The prizes will consist of gold medals, and the competing essays are to be given to the Secretary of the Society by the end of January in each year.—*New York Medical Record.*

**Health of the Chinese.**—The Chinese consul in New York states that, despite the apparent neglect by the Chinese of most laws that to our ways of thinking are absolutely essential to health, it is rare that one of the race dies of a zymotic disease. He says that his people have been studying the laws of health for the last thousand years, and that they have, to this extent, mastered those laws is proved, to his mind, by the circumstance that contagious disease is seldom found among them.—*Medical Times.*

**New Method of Treating Gonorrhœa.**—Senfltleren (Monatsh. f. prakt. Dermatol.) used small medicated bougies, which he introduced into the urethra by means of an instrument that he calls a "pistol" (probably similar to a Sims' uterine applicator) pushing them down to the membranous portion, where they are left to dissolve. The first application is said to be very painful, but the sensitiveness soon disappears. The following is the formula for one variety of these bougies :

| | | |
|---|---|---|
| ℞. | Subnitrate of bismuth, white of egg, each..... | 15 grains. |
| | Gum Arabic.................................. | 45 grains. |
| | Glycerin .................................. | 5 drops. |
| | Mucilage.................................. | 1 drop. |

Make 10 bougies, from 7 to 10 ctm. long.

Iodoform, sulpho-carbonate of zinc, corrosive sublimate, and nitrate of silver are also used, in amounts varying from one-sixteenth of a grain (of the sublimate) to two grains. The following advantages are alleged : 1. The application is strictly localized at the seat of the inflammation, the exact situation of which may be determined precisely by means of the endoscope. 2. The medicinal substance remains in contact with the inflamed surface for a long time, which is not the case when injections are given. 3. The inflammation is cut short, so that the danger of stricture is averted. The writer is not satisfied with pursuing the treatment until all discharge has ceased, but continues it until not a single sensitive spot remains in the urethra.—*N. Y. Medical Journal.*

**Water for Infants.**—A physician of the New York Nursery and Child's Hospital believes, from his practice, that infants generally, whether brought up at the breast or artificially, are not supplied with sufficient water, the fluid portion of their food being quickly taken up, and leaving the solid too thick to be easily digested. In warm, dry weather, healthy babies will take water every hour with advantage, and their frequent fretfulness and rise of temperature is often directly due to their not having it. A free supply of water, and restricting the frequency of nursing, has been found at the nursery to be a most effectual check in cases of incipient fever, a diminished rate of mortality and marked reduction in the number of gastric and intestinal complaints being attributed to this cause. In teeth-cutting, water soothes the gums, and frequently stops the fretting and restlessness universal in children at this period.—*Southern Journal Health.*

**Vaccination with Fever Virus.**—The following telegram comes from the city of Mexico : "The Government of Mexico has permitted the garrison of Vera Cruz to be vaccinated with yellow-fever virus, according to Dr. Carmona's system. Experiments were first made on prisoners who volunteered for the purpose. Persons vaccinated with the virus have all the premonitory symptoms of the fever. It is thought that the inoculation will serve as a complete protection for four or five years. Great interest is felt in the discovery, and the system will be tried on the west coast and in Sonora."

